# Authors' Reply

**DOI:** 10.1371/journal.pmed.0030127

**Published:** 2006-02-28

**Authors:** Valeriya Lyssenko, Dragi Anevski, Peter Almgren, Leif Groop

**Affiliations:** **1**Lund UniversityMalmöSweden

In their correspondence to our paper, “Genetic Prediction of Type 2 Diabetes” [ 1], Janssens et al. question whether genetic testing really will improve prediction of future type 2 diabetes (T2D) [ 2].

Their criticism is well taken and important for the discussion on the causes of T2D. It is assumed, although not yet proven, that T2D results from the complex interaction between multiple genetic variants and environmental risk factors. In an earlier paper [ 3], we listed three of those risk factors: family history of T2D, body mass index (BMI) greater than or equal to 30 kg/m ^2^, and non-normal fasting plasma glucose (FPG) greater than 5.5 mmol/l. Two of these risk factors (BMI and FPG) were replicated in a study of Israeli military men [ 4]. If we assume that T2D results from the interaction between these risk factors and genetic variants, it is not unreasonable to test whether genetic risk factors improve the predictive value of the environmental risk factors.

Therefore, the main objective of our analysis was to find a joint genetic and environmental model that accurately describes the effects on the time distribution of the age at onset, and to use that model to quantify the genetic and environmental effects. For that purpose, we have performed forward stepwise multivariate Cox regression analysis, adding one covariate at a time. The resulting multivariate model has then been tested against a “null” model with a likelihood-ratio test to assess the overall fit of the model. To address the question asked in the introduction, we choose to study the effects of having all the significant risk factors elevated against the effects of having none by using the estimates in the resulting model. This approach is open for discussion; one alternative approach would be the approach presented by Janssens et al.

They make a group division and use logistic regression to assess genetic effects on the development of T2D. There are two drawbacks with this approach. The first is that by dividing the population into subgroups one lowers the power to find significant effects and also obtains large variances in the estimated effects, thus getting a large degree of uncertainty in both significance and size of the estimated effects. Secondly, logistic regression disregards the fact that individuals have been followed prospectively. In contrast, we perform calculations of estimated effects in the obtained multivariate model, thereby using all the available data in the variance calculations. This allows us to assess the size of an increment of a risk factor for prediction of the disease, e.g., the estimated hazard ratio (HR) for a continuous covariate is the ratio of the hazards for an increase of one in the covariate.

Using our multivariate model, the genetic factors confer a T2D risk of the same magnitude (HR, 3.69) as, e.g., FPG (HR, 3.25) and BMI (HR, 1.77).

Janssens et al. claim that a receiver-operating characteristic (ROC) analysis on our data does not support the claim that there is significant gene effect for discriminative purposes. However, Janssens et al. perform an ROC and an area under the curve (AUC) analysis based on a logistic regression, thus treating each individual as affected versus nonaffected and not taking into consideration age effects, the drawbacks of which are discussed above.

An ROC analysis may not be the best way to describe modest effects of genetic variants contributing to risk of a polygenic disorder such as T2D. This is further hampered by the use of longitudinal data with several time points. Although the ROC analysis recently [ 5] has been applied to survival analysis data, the complexity of the situation in T2D limits its value. Using this approach, we obtain an AUC of 0.76 for the full model and an AUC of 0.75 for the model excluding genetic effects.

However, an ROC analysis could work for genetic prediction of a monogenic disorder such as maturity-onset diabetes of the young (MODY). MODY 3 is a dominant form of early-onset diabetes with strong penetrance, and is caused by mutations in the hepatocyte nuclear factor 1, *HNF-1*α gene [ 6]. In an analysis of 33 carriers of MODY mutations followed for a mean of 4.7 years, the AUC for diagnosis of MODY was 0.86.

We should keep in mind that we have only tested a few potential common genetic variants contributing to T2D. It was recently suggested that only approximately 20 genes may be needed to explain 50% of the disease burden in the population [ 7]. However, for individual genetic prediction, we need to establish how much of the relative risk (λs) is accounted for by genetic variants. For this purpose, we need to know all variants which contribute to risk of T2D. Hopefully, genome-wide single nucleotide polymorphism (SNP) scans in the future can provide us with that information.
